# Recurrent Ptosis in a Case of Dubowitz Syndrome

**DOI:** 10.7759/cureus.16436

**Published:** 2021-07-17

**Authors:** Sahil Agrawal, Arpita Kulshrestha, Deepsekhar Das, Mandeep S Bajaj, Sujeeth Modaboyina

**Affiliations:** 1 Ophthalmology, All India Institute of Medical Sciences, New Delhi, IND

**Keywords:** mental retardation, dubowitz, recurrent ptosis, autosomal recessive, craniofacial abnormality, eczema

## Abstract

Dubowitz syndrome is a relatively rare genetic and developmental disorder. An eight-year-old female presented with a complaint of drooping in her left eye since birth. She had undergone ptosis surgery two years back. There was a history of delayed speech and delayed dentition. She was of moderate built appropriate to her age. There was microcephaly, sparse hair, flat bridge of the nose with a prominent rounded tip, short stature, low-set ears, and micrognathia with subsequent protrusion of upper two incisors. Based on the clinical features a diagnosis of Dubowitz syndrome with left recurrent ptosis was made. She underwent frontalis sling surgery and had a satisfactory outcome.

## Introduction

Dubowitz syndrome is a relatively rare genetic and developmental disorder of a presumed but an unknown genetic cause. It is an autosomal recessive disorder, including intrauterine and postnatal growth retardation, craniofacial abnormalities, mental retardation, and eczematous skin eruption [1]. We herein report the case of a child with Dubowitz syndrome with recurrent ptosis.

## Case presentation

**Figure 1:**
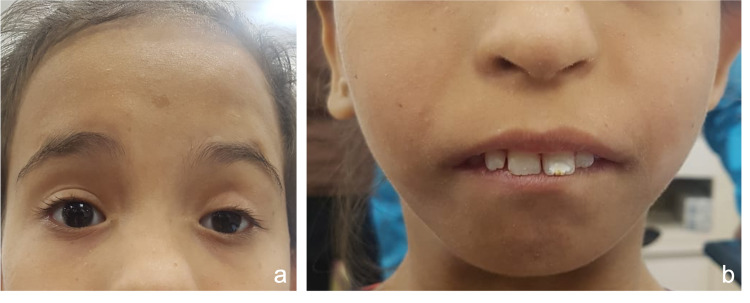
a. Clinical photograph of the patient showing microcephaly with sparse hairs with blepharophimosis, frontalis overaction, and ptosis in the left eye. b. Clinical photograph showing low-set ears, flat bridge of nose with a prominent rounded tip, micrognathia, and protruded upper incisors.

An eight-year-old female presented with complaints of drooping in her left eye since birth. The doctor then had advised that she would be taken up for necessary surgery later on after completion of facial growth. The parents gave a history of the child undergoing a ptosis surgery two years back, following which there was limited correction; however, the drooping persisted. There was a history of delayed speech and delayed dentition with other developmental milestones achieved timely. On examination, there was microcephaly, sparse hair, flat bridge of the nose with a prominent rounded tip, short stature, low-set ears, and micrognathia with subsequent protrusion of upper two incisors (Figure 1a, b). She has a normal growth appropriate to her age.

On ophthalmological examination, blepharophimosis was noted as the horizontal palpebral apertures measured 28 mm on the right and 27 mm on the left side (Figure 1a). There was bilateral overaction of the frontalis muscle with arching of the eyebrows. There was bilateral simple congenital ptosis with mild ptosis in the right side and severe in the left (Figure 1a). There was a horizontal scar on the left upper eyelid hinting towards a previous levator palpebralis superioris surgery in the past. The levator excursion in the right was 8 mm and 3 mm in the left. The ocular surface was healthy; there was no epithelial defect or surface keratinization. The rest of the ophthalmic examination was within normal limits. Based on the clinical features, a diagnosis of Dubowitz syndrome with left recurrent ptosis was made.

After an explained informed consent, she was taken up for a frontalis sling surgery. Under general anaesthesia, conventional Fox pentagon was carried out via a total of five incisions - two in the upper eyelid and three supra-brow incisions. Post-operatively she had a satisfactory outcome with MRD1 in both eyes similar.

## Discussion

Dubowitz syndrome was first described by Dubowitz in 1965, and the phenotype was later elaborated in 1971. It seems to have a wide range of phenotypic variability. The facial appearance is considered to be the most diagnostic manifestation of the syndrome and is strikingly similar in all patients. Typical craniofacial features include microcephaly, sparse hair, a high forehead, wide nasal bridge, palate anomalies, micrognathia/retrognathia, and dysplastic ears. Ophthalmologic findings seen in about 20% of patients included telecanthus (60%), ptosis/blepharophimosis (often asymmetrical) (65%), epicanthal folds (50%), and strabismus [1]. Other ocular abnormalities, such as microphthalmos, coloboma, and total cataract, have been reported [2].

A typical symptom is eczema of the lower face and the flexures of elbows and knees. Mild-to-moderate mental retardation is observed in about 50% of patients. Abnormal behaviour and hyperactivity have been described in 40% of patients [2]. Other behavioural abnormalities, such as minimal attention, aggressiveness, shyness, dislike of crowds, food refusal and bed-wetting, have all been reported [3]. Further findings include cryptorchidism in the affected males, brachy-clinodactyly of the fifth finger, and susceptibility to tumour formation. While life-threatening anomalies are rare in this syndrome, the two important causes of morbidity and mortality are malignancy (haematological) and bone marrow failure [4].

A study reported the NSUN2 gene as the first potential causal gene with a relationship to the Dubowitz spectrum phenotype [5]. Some studies indicate that LIG4 can be responsible for the disorder [6], while others suggested deletions at Ch14q32 to have phenotype overlap with Dubowitz syndrome [7]. The existence of an equal sex ratio and association of parental consanguinity suggested an autosomal recessive pattern of inheritance [5].

Dubowitz syndrome shares symptoms of mental and growth retardation, chromosomal instability, and skin lesions with other disorders such as Bloom syndrome, Fanconi anaemia, foetal alcohol syndrome, and Seckel syndrome. The main feature that distinguishes Dubowitz syndrome from Bloom syndrome and Fanconi is the facial appearance. Also, Bloom syndrome characteristically has telangiectatic erythema on the face. Foetal alcohol syndrome presents with comparable facial features but has minimal telecanthus, neural tube defect, absence of eczema, and a history of prenatal alcohol exposure [8]. The mental retardation in Seckel syndrome may be more severe with distinct facial features such as a “beak-like” protrusion of the nose. We also need to differentiate from other eyelid anomaly syndromes like Frydman-Cohen syndrome and Floating-Harbour syndrome [9,10].

## Conclusions

Although Dubowitz syndrome has been thoroughly described in the literature, this is the first time that recurrent ptosis has been reported. Given the limited data on the condition, this finding may be a new feature to add to the clinical presentation of Dubowitz syndrome.
